# Physical function and lean body mass as predictors of bone loss after hip fracture: a prospective follow-up study

**DOI:** 10.1186/s12891-020-03401-3

**Published:** 2020-06-09

**Authors:** Tuuli H. Suominen, Johanna Edgren, Anu Salpakoski, Mauri Kallinen, Tomas Cervinka, Timo Rantalainen, Timo Törmäkangas, Ari Heinonen, Sarianna Sipilä

**Affiliations:** 1 Gerontology Research Center, Faculty of Sport and Health Sciences, University of Jyväskylä, PO Box 35, 40014 Jyväskylä, Finland; 2 Rehabilitation Center Peurunka, Laukaa, Finland; 3 Department of Physical and Rehabilitation Medicine, Central Finland Health Care District, Central Finland Central Hospital, Jyväskylä, Finland; 4 Center for Life Course Health Research, University of Oulu, Oulu, Finland; 5 Satakunta University of Applied Sciences, Pori, Finland; 6 Faculty of Sport and Health Sciences, University of Jyväskylä, Jyväskylä, Finland

**Keywords:** Aging, Bone mineral density, Hip fracture, Lean body mass, Physical function, pQCT

## Abstract

**Background:**

Predictors of bone deterioration after hip fracture have not been well characterized. The aim of this study was to examine the associations of physical function and lean body mass (LBM) with loss of bone density and strength in older people recovering from a hip fracture.

**Methods:**

A total of 81 over 60-year-old, community-dwelling men and women operated for a hip fracture participated in this 1-year prospective follow-up study. Distal tibia total volumetric bone mineral density (vBMD_TOT_, mg/cm^3^) and compressive strength index (BSI, g^2^/cm^4^) and mid-tibia cortical vBMD (vBMD_CO_, mg/cm^3^) and bending strength index (SSI, mm^3^) were assessed in both legs by peripheral quantitative computed tomography (pQCT) at baseline (on average 10 weeks after fracture) and at 12 months. At baseline, LBM was measured with a bioimpedance device and physical function with the Short Physical Performance Battery (SPPB) and perceived difficulty in walking outdoors. Robust multivariable linear regression models were used to estimate the associations of physical function and LBM with the change in bone parameters at 12-months.

**Results:**

The mean change in distal tibia vBMD_TOT_ and BSI in both legs ranged from − 0.9 to − 2.5%. The change in mid-tibia vBMD_CO_ and SSI ranged from − 0.5 to − 2.1%. A lower SPPB score, difficulty in walking outdoors and lower LBM predicted greater decline in distal tibia vBMD_TOT_ in both legs. A lower SPPB score and difficulty in walking outdoors were also associated with a greater decline in distal tibia BSI in both legs. At the midshaft site, a lower SPPB score and lower LBM were associated with greater decline in SSI on the fractured side.

**Conclusions:**

Older hip fracture patients with low physical function and lower LBM may be at risk for greater decline in tibia bone properties during the first post-fracture year. Acknowledgement of the risk factors could assist in developing interventions and care to promote bone health and overall recovery.

**Trial registration:**

ISRCTN, ISRCTN53680197. The trial was registered retrospectively but before the recruitment was completed. Registered March 3, 2010.

## Background

Substantial decrements in physical function and muscle occur after hip fracture [1, 2], and less than half of these patients recover their pre-fracture level of function [3, 4]. In addition, hip fracture is followed by accelerated and long-term decline in bone structure, density and strength [5–9], especially in the leg on the fractured side [6, 10]. Together with the loss of physical function, bone deterioration increases the risk for a subsequent fracture [11, 12]. Post-hip fracture bone loss can probably be explained in part by disuse, but to date systematic exploration of the factors contributing to post-fracture bone deterioration has been rather scarce.

Low level of physical function is a risk factor for poorer recovery after hip fracture [13]. With the present study population, we have previously shown better recovery of physical function in patients with less difficulty in their post-discharge ability to walk outdoors [13]. A low level of physical function may also prevent effective loading of the bones and could be related to reduced bone-loading physical activity. Moreover, owing to the strong relationship between bone and muscle [14], lower LBM could also contribute to the increase in post-hip fracture bone loss. The positive relationship between bone and muscle can be traced to several biochemical (myokines and osteokines) and biomechanical factors, including gravitational loading on weight-bearing bones and the associated effect of muscle contraction [15], which places the greatest load on bones [14]. In older men, higher LBM has also been associated with better functional recovery after hip fracture [16].

The few studies that have explored the factors contributing to bone loss after hip fracture [2, 17] have not reported either measures of bone strength or outcomes for the leg on the fractured side. The aim of this study was to examine whether physical function, measured with SPPB and perceived difficulty in walking outdoors, and LBM predict the change at 12 months in the bone density and strength of both legs in older men and women recovering from a recent hip fracture.

## Methods

### Design and participants

This study utilizes data from a randomized controlled trial (ISRCTN53680197) investigating the effects of a yearlong home-based rehabilitation program compared to standard care on mobility recovery among over 60-year-old, ambulatory and community-dwelling older people with a recent hip fracture [18]. The design and recruitment procedure have been reported in detail earlier [19]. Briefly, all men and women who had been operated for a femoral neck or pretrochanteric fracture (ICD code S72.0 or S72.1) in the Central Finland Central Hospital (Jyväskylä, Finland) between 1.3.2008 and 31.12.2010, and fulfilling the inclusion criteria were informed about the study (*n =* 269). Of these, 161 were interested and further informed on the study. After preliminary assessment of eligibility, 136 persons were invited to the baseline measurements. Patients suffering from severe memory problems (Mini Mental State Examination, MMSE < 18), alcoholism, a severe cardiovascular or pulmonary condition or some other progressive disease, and severe depression (Beck Depression Inventory BDI-II > 29) were excluded from the study. Thereafter, 81 eligible patients participated in the study.

After the baseline measurements, conducted on average 10 weeks post fracture, the participants were randomized into an intervention (*n* = 40) and a standard care control (*n* = 41) group using a computer-generated group allocation list generated by a blinded statistician, who was not involved in either the recruitment or data collection process. Blocks of 10, stratified by gender and surgical procedure (internal fixation vs arthroplasty), were used. Follow-up measurements were arranged at 3, 6, and 12 months after the baseline measurements. All assessments were conducted at the research laboratory, and all outcome assessors were blinded to the treatment-group assignment. For the present analyses, data from the rehabilitation and standard care control groups were pooled, since the intervention had no effect on bone properties [10], and only baseline and 12-month follow-up bone data were utilized.

The sample size calculations have been reported in detail before [18, 19]. Briefly, an a priori sample size calculation was performed for the primary outcome, mobility limitation, based on previously published longitudinal data on mobility recovery after a hip fracture [20]. Based on calculations, a minimum of 44 participants were needed in each group (in total 88 participants) to detect the expected difference between the study groups at a level of significance of α = 0.05 and β = 0.20. Sample size was calculated using an online sample size calculator (DSS researcher’s toolkit).

### Intervention and control condition

All participants received standard care from the hospital. In addition, the intervention group received a year-long, physical rehabilitation program aimed at restoring mobility and physical functional capacity to the pre hip fracture level [18, 19]. The individually tailored program comprised an evaluation and modification of environmental hazards, guidance for safe walking, non-pharmacological pain management, motivational physical activity counselling and a progressive home exercise program. The intervention took place in the participants’ homes and included five to six home visits by a physiotherapist. The progressive home exercise program comprised strengthening exercises for the lower limb muscles using resistance bands, balance training in the standing position, stretching, and functional exercises including walking, reaching, turning in different directions and stair climbing [10, 18].

### Peripheral quantitative computed tomography (pQCT)

Bone scans from the distal tibia (5% of measured tibial length proximal to the distal end plate) and tibial shaft (55%) of both legs were obtained by pQCT (XCT-2000, Stratec Medizintechnik, Pforzheim, Germany) according to methods described earlier [10]. The pQCT scans were analyzed with an automated threshold-free cortical bone detection method [the outer boundary detection and subsequent shrinking (OBS) procedure, OBS cortical bone detection 2.1] [21, 22]. The outcome variables for the distal tibia were total volumetric bone mineral density (vBMD_TOT_, mg/cm^3^) and compressive bone strength index (BSI, g^2^/cm^4^ = vBMD_TOT_^2^ × CSA_TOT_) [8, 9]. For the midshaft site, the variables were cortical vBMD (vBMD_CO_) and a strength-strain index (SSI, mm^3^; density-weighted polar section modulus) reflecting the bone’s resistance to bending and torsional loads. The root mean square coefficient of variation (CV_RMS_) for the BMD and strength index measurements in our laboratory ranges from 0.4 to 1.6% [23].

### Physical function

Physical function at baseline was measured using the Short Physical Performance Battery (SPPB) test, which includes habitual walking speed, chair rise and standing balance tests [24]. A higher score (range, 0–12) indicates better performance. Perceived difficulty in walking outdoors was assessed by a questionnaire with the following response categories: 1) no difficulties, 2) some difficulties, 3) a great deal of difficulties, 4) manage only with help, and 5) unable to manage even with help.

### Lean body mass

Lean body mass (kg) was assessed with a bioimpedance device with eight polar electrodes (BC-418; TANITA, Tokyo, Japan). Participants were instructed to avoid caffeine for 2 h, alcohol for 36 h and physical exercise for 24 h before testing.

### Health, fracture status and anthropometry

The presence of chronic conditions, use of prescription medications, fracture date and status, and type and date of surgery were assessed by means of a pre-structured questionnaire, current prescriptions and medical records and confirmed in a medical examination performed by a research nurse and a physician. Contraindications for the physical performance assessments were evaluated according to ACSM guidelines [25]. Body height and weight were measured using standard procedures, and body mass index was calculated as body weight divided by height squared (kg/m^2^). Fat percentage was assessed with bioimpedance device. Serum concentrations of 25-hydroxyvitamin D (25OHD, nmol/L) and parathyroid hormone (PTH, ng/L) were determined according to methods described earlier [10]. Smoking status was assessed by questionnaire and categorized as current, former and never smokers.

### Statistical analysis

Mean values and standard deviations (SD) were calculated using standard procedures. To alleviate problems resulting from extreme outliers, we used the robust linear regression approach [26] to estimate the associations of the predictor variables with each dependent variable. The mean percentage changes in vBMD and the bone strength indices, used as outcome variables, were calculated as [(follow-up – baseline)/baseline × 100]. Baseline LBM, SPPB score and ability to walk outdoors were entered in the models at the same time as predictors. The SPPB scores were recoded into a single binary variable: 0) high performance (score ≥ 7) or 1) low performance (score < 7). A score below 7 indicates a high risk for disability [27]. The categories of perceived difficulty in walking outdoors were recoded as 0) major difficulties or unable (categories 3–5), or 1) no difficulties or minor difficulties (categories 1–2). Predictive mean matching of the ‘mice’ package [28] in the R programming environment was used to impute missing values in LBM for three subjects. The models were adjusted for potential confounders: age, gender, surgical procedure (internal fixation vs hemiarthroplasty vs total arthroplasty), number of chronic diseases and use of bisphosphonate medication (yes/no) at baseline. The study group was not included in the models, as no differences were observed in baseline characteristics between the groups and the intervention had no effect on bone properties [10]. The main reasons for missing bone data were inability to perform the measurements, inaccurate positioning of the leg, a technically invalid pQCT scan, substantial movement artifacts, and metal in tissues in the scanned region. For the distal tibia, a total of 154 valid scans were obtained at baseline, and 130 at 12 months. For the midshaft site, the corresponding numbers were 156 and 130. Descriptive analyses were performed using SPSS 24.0 software (IBM, NY, USA) and the robust linear regression models were analyzed using R version 3.5.1 (R core team, Vienna, Austria) with the significance level set at 5%. The study power, calculated for the main outcome, mobility limitation, was 78%.

## Results

Baseline characteristics of the participants are shown in Table 1. During the 12-month study, three participants dropped out for personal reasons, and one participant died unrelated to the research procedures. The mean change from baseline to 12 months in distal tibia vBMD_TOT_ was − 1.5 (SD 4.6) % on the fractured and − 0.9 (3.7) % on the non-fractured side. The corresponding changes for BSI were − 2.1 (8.8) % and − 2.5 (7.0) %, respectively. At the midshaft site, the mean change in vBMD_CO_ was − 1.3 (2.3) % on the fractured and − 0.5 (1.6) % on the non-fractured side. The corresponding changes for SSI were − 2.1 (4.4) % and − 1.5 (3.4) %.

**Table 1:** Baseline characteristics of the participants

	*n*	Mean (SD) or *n* (%)
Age, years	81	80.0 (7.1)
Women, *n* (%)	81	63 (78)
Height, cm	80	161 (9)
Weight, kg	81	65.8 (11.5)
Body mass index, kg/m^2^	80	25.5 (3.8)
Body fat, %	76	31.3 (6.5)
Lean body mass, kg	76	44.5 (8.4)
Smoking, n (%)	81	
Never		64 (79)
Former		10 (12)
Current		7 (9)
Number of chronic diseases	81	3 (2)
Current bisphosphonate use, *n* (%)	81	16 (20)
Serum-25OHD, nmol/L	68	55 (23)
Serum-PTH, ng/L	68	49 (23)
Time since fracture (days)	81	70 (28)
Site of fracture, n (%)	81	
Femoral neck		52 (64)
Pertrochanteric		29 (36)
Type of surgery, *n* (%)	81	
Internal fixation		38 (47)
Hemiarthroplasty		33 (41)
Total arthroplasty		10 (12)
Physical function		
SPPB score (range, 0–12)	81	6.2 (2.4)
SPPB score < 7, *n* (%)		42 (52)
Walking outdoors, *n* (%)	81	
No difficulties		12 (15)
Some difficulties		38 (47)
Great deal of difficulties		13 (16)
Manage only with help		17 (21)
Unable to manage even with help		1 (1)
Distal tibia		
vBMD_TOT_fractured leg_ (mg/cm^3^)	76	215 (52)
BSI_fractured leg_ (g^2^/cm^4^)	76	0.50 (0.25)
vBMD_TOT_non-fractured leg_ (mg/cm^3^)	78	218 (52)
BSI_non-fractured leg_ (g^2^/cm^4^)	78	0.51 (0.25)
Tibial midshaft		
vBMD_CO_fractured leg_ (mg/cm^3^)	78	1043 (71)
SSI__fractured leg_ (mm^3^)	78	1493 (453)
vBMD_CO_non-fractured leg_ (mg/cm^3^)	78	1045 (78)
SSI__non-fractured leg_ (mm^3^)	78	1516 (450)

*SPPB* Short Physical Performance Battery, *vBMD*_*TOT*_ Total volumetric bone mineral density, *BSI* Compressive bone strength index, *vBMD*_*CO*_ Cortical vBMD, *SSI* Strength-strain index

In the adjusted multivariable regression analyses, a lower SPPB score, difficulty in walking outdoors and lower LBM at baseline predicted greater decline in distal tibia vBMD_TOT_ both on the fractured and non-fractured sides (Table 2). A lower SPPB score and difficulty in walking outdoors were also predictive of a greater decline in the distal tibia BSI on both sides. At the midshaft site, a lower SPPB score and lower LBM were associated with a greater decline in the SSI on the fractured side.

**Table 2:** Multiple linear regression models predicting changes in distal tibia and tibial mid-shaft bone characteristics

	Fractured side				Non-fractured side			
**Distal tibia**	**vBMD**_**TOT**_ (*n* = 58)		**BSI** (*n* = 58)		**vBMD**_**TOT**_ (*n* = 59)		**BSI** (*n* = 56)	
	B (SE)	*p*	B (SE)	*p*	B (SE)	*p*	B (SE)	*p*
Lean body mass	.152 (0.06)	.010	.136 (0.12)	.258	.151 (0.06)	.010	.152 (0.10)	.126
SPPB^a^	−1.44 (0.64)	.028	−3.09 (1.33)	.023	−1.34 (0.64)	.040	−3.06 (1.11)	.007
Walking outdoors^b^	−2.05 (0.74)	.009	−3.62 (1.53)	.024	−2.67 (0.74)	<.001	−5.69 (1.28)	<.001
	*R*^2^ = .236	<.001	*R*^2^ = .182	.006	*R*^2^ = .363	<.001	*R*^2^ = .428	<.001
**Tibial midshaft**	**vBMD**_**CO**_ (*n* = 57)		**SSI** (*n* = 58)		**vBMD**_**CO**_ (*n* = 58)		**SSI** (*n* = 57)	
	B (SE)	*p*	B (SE)	*p*	B (SE)	*p*	B (SE)	*p*
Lean body mass	−.025 (0.04)	.531	.171 (0.08)	.042	−.004 (0.04)	.910	.129 (0.07)	.067
SPPB^a^	−.538 (0.46)	.247	−2.03 (0.93)	.034	−.193 (0.43)	.655	−.062 (0.78)	.938
Walking outdoors^b^	−.371 (0.54)	.485	−0.60 (1.08)	.578	−.356 (0.50)	.476	−.343 (0.90)	.711
	*R*^2^ = .119	.286	*R*^2^ = .247	.023	*R*^2^ = .015	.566	*R*^2^ = .157	.421

The models were adjusted for age, sex, surgical procedure, number of chronic diseases and use of bisphosphonates at baseline. Sample size reduction due to weighting ranged from 5 to 10%

*vBMD*_*TOT*_ Total volumetric bone mineral density, *BSI* Compressive bone strength index, *vBMD*_*CO*_ Cortical vBMD, *SSI* Strength-strain index, *SPPB* Short Physical Performance Battery

^a^0) high performance (score ≥ 7), 1) low performance (score < 7)

^b^0) without difficulties/minor difficulty 1) major difficulty/unable

## Discussion

In this 12-month follow-up study of older, community-dwelling men and women operated for a hip fracture, we found that lower physical function and lower LBM predicted greater decline in distal tibia bone density during the year following the fracture. Lower physical function was also associated with a greater decline in distal tibia bone strength. At the midshaft site, a lower SPPB score and lower LBM were predictive of a greater decline in bone strength on the fractured side.

Despite the large body of research on the factors contributing accelerated bone loss in aging people, very few have been conducted on hip fracture patients [2, 17] and none have examined potential predictors of the changes in bone properties over time. As in our previous studies [6, 10], bone deterioration was more pronounced in the leg on the fractured side, a finding which may partly be explained by disuse. No between-side differences in the predictors were, however, found except for mid-tibia SSI. At the midshaft site, a lower SPPB score and lower LBM were predictive of greater decline in the SSI on the fractured side only. Neither the decline in SSI on the non-fractured side nor the decline in cortical vBMD on both sides was associated with any of the factors studied, suggesting that the mid-tibia may not be equally sensitive to differences in the predictors used.

In the present study, a lower SPPB score (under 7) and major difficulty in the ability to walk outdoors predicted greater deterioration in distal tibia volumetric bone density and strength. This is in line with our previous findings indicating better functional recovery in patients with better function [13] and supports our hypothesis of better bone recovery in patients with a better capacity to load their bones. In old age, walking outdoors has also been associated with a greater amount of objectively measured physical activity [29]. Furthermore, our results are in line with previous studies suggesting better post-fracture functional recovery [16] and reduced age-related bone loss [30] for men with higher LBM. Moreover, in older, often frail and undernourished, hip fracture patients, higher LBM may also reflect better resources to cope with a prolonged catabolic state and the hip fracture-related stresses.

This study has its limitations. The study reports the results of a secondary analysis of an RCT, and the observational design demonstrates only associations, not causal relationships. Because of missing data and a relatively small sample size, we had to limit the number of possible confounders included in the analyses. Down-weighting the influence of outliers in the regression analyses further reduced the sample size but yielded more reliable regression coefficients. Moreover, the participants were community-living, and therefore the results may not be generalizable to all hip fracture patients. Furthermore, DXA or MRI would have provided more accurate LBM results. Finally, constraints related to the imaging method used, such as scan resolution, partial volume effect and beam hardening should be considered when interpreting the results. The strengths of the study include a population-based clinical study sample, a 3D imaging modality to assess changes in volumetric bone mineral density and estimated strength in the leg on the fractured side, and a longitudinal follow-up of sufficient length to detect changes in bone properties.

## Conclusions

In conclusion, low physical function and lower LBM may increase the risk for accelerated bone deterioration in older hip fracture patients. Attention should be paid to patients at greater risk for poorer recovery, and more effective, multidimensional and individualized interventions and care should be provided to promote bone health and overall recovery. Due to limited possibilities to prevent bone deterioration after hip fracture, attention should be paid to physical function, muscle mass preservation and fall prevention before as well as after fracture occurrence.

## Data Availability

The datasets used and analyzed during the current study are available from SS on reasonable request.

## Abbreviations

25OHD: 25-hydroxyvitamin D
BSI: Compressive bone strength index
LBM: Lean body mass
OBS: Outer boundary detection and subsequent shrinking procedure
pQCT: Peripheral quantitative computed tomography
PTH: Parathyroid hormone
SPPB: Short Physical Performance Battery
SSI: Strength-strain index
vBMD_CO_: Cortical volumetric bone mineral density
vBMD_TOT_: Total volumetric bone mineral density
